# *BCR‐ABL1* transcript levels at 4 weeks have prognostic significance for time‐specific responses and for predicting survival in chronic‐phase chronic myeloid leukemia patients treated with various tyrosine kinase inhibitors

**DOI:** 10.1002/cam4.1753

**Published:** 2018-08-31

**Authors:** Hye‐young Song, Hayeon Noh, Soo young Choi, Sung‐Eun Lee, Soo‐Hyun Kim, Kyung‐Mi Kee, Hea‐Lyun Yoo, Mi‐young Lee, Ki‐Hoon Kang, Ji‐Hyung Suh, Seon‐young Yang, Eun‐Jung Jang, Jangik I. Lee, Dong‐Wook Kim

**Affiliations:** ^1^ Leukemia Research Institute The Catholic University of Korea Seoul Korea; ^2^ Department of Pharmacy College of Pharmacy Yonsei University Incheon Korea; ^3^ Department of Hematology Seoul St. Mary's Hospital The Catholic University of Korea Seoul Korea; ^4^ Department of Pharmacy Seoul National University Hospital Seoul Korea

**Keywords:** chronic myeloid leukemia, early molecular response, molecular response, predictor, tyrosine kinase inhibitor

## Abstract

The present study aimed to assess the clinical impact of *BCR‐ABL1* transcript levels determined at an earlier time point than the 3‐month early molecular response (EMR) in chronic‐phase chronic myeloid leukemia (CML‐CP) patients. *BCR‐ABL1* transcript levels of CML‐CP patients (n = 258; median age, 43 [range, 18‐81] years) treated with various tyrosine kinase inhibitors (TKIs) were determined at 4 weeks (28 ± 3 days) and at every 3 months of treatment initiation. At 4 weeks, receiver operating characteristic curves revealed that cutoff values of *BCR‐ABL1* transcripts for achieving major molecular responses (MMRs) by 12 and 60 months were 40.89% and 39.16%, respectively (95% CI, 0.658‐0.772 and 95% CI, 0.643‐0.758; *P *<* *0.0001). With 40% of *BCR‐ABL1* transcripts at 4 weeks (very early MR; VEMR), patients with VEMR achieved higher 3‐month EMR and 4‐week VEMR significantly associated with higher cumulative incidences of 5‐year MMR (89.1% vs 72.3%; *P *<* *0.001) and 5‐year deep molecular response (DMR) (56.5% vs 29.4%; *P *=* *0.001). Furthermore, event‐free survival (EFS)‐a (93.0% vs 84.8%; *P *=* *0.068) and EFS‐b (71.1% vs 57.9%; *P *=* *0.061) by 5 years were also marginally significant. VEMR and 3‐month EMR were achieved in 89 patients, with significantly superior outcomes. In multivariate analyses, lower leukocyte count (*P *=* *0.008) and frontline second‐generation TKI therapy size (*P *<* *0.001) were significantly associated with VEMR achievement, but not baseline *BCR‐ABL1* level and CML duration. In conclusion, the 4‐week *BCR‐ABL1* transcript levels including VEMR could be important to predict long‐term outcomes and may provide additional information about innate intrinsic sensitivity to CML among individuals.

## 1 INTRODUCTION

Delayed cytogenetic and molecular responses are associated with increased tumor progression and poor outcome in imatinib‐treated patients with newly diagnosed chronic‐phase chronic myeloid leukemia (CML‐CP).^1^, ^2^, ^3^ Early molecular response (EMR; *BCR‐ABL1* ≤10% at 3 months, ≤1% at 6 months) predicts long‐term response and survival.^4^, ^5^, ^6^ Therefore, EMR achievement at an early time point of treatment has been reported as an important parameter for further treatment and monitoring.^7^, ^8^, ^9^

Hughes et al^4^ reported that patients achieving EMR at 6 months had superior event‐free survival (EFS) and decreased tumor progression. Moreover, numerous studies including those on second‐generation (2G) tyrosine kinase inhibitors (TKIs) revealed that patients achieving EMR had significantly lower overall survival (OS), progression‐free survival (PFS), complete cytogenetic response (CCyR), major molecular response (MMR), and/or complete molecular response (CMR).^5^, ^10^, ^11^, ^12^, ^13^, ^14^ The prognostic information from 3‐month vs 6‐month EMR achievement has been analyzed, and earlier achievement of EMR is reported to have superior prognostic value.^6^, ^15^, ^16^ In addition, as various factors such as baseline risk score, spleen size, baseline leukocyte count, TKI dose intensity, and blood TKI levels can be associated with EMR achievement, more cautious analyses are warranted.^13^, ^17^

To investigate prognostic significance using molecular data at earlier time points, recent studies highlighted that 3‐month EMR depends on the baseline leukemia burden and a decline in *BCR‐ABL1* transcripts.^10^, ^18^, ^19^ Hanfstein et al^10^ found that absolute *BCR‐ABL1* transcript level at diagnosis was not predictive, and an individual reduction to the half‐log of the baseline level (0.35‐fold; 0.46 log) at 3 months significantly discriminated for 5‐year OS and PFS. Moreover, the rate of *BCR‐ABL1* decline as assessed by halving time was a critical predictor for very poor outcomes among non‐EMR patients at 3 months.^18^ Additional studies have reported that the halving time was an important predictor for the achievement of molecular responses and was shorter in patients administered frontline 2G TKIs.^19^, ^20^, ^21^ Recently, El Missiry et al^22^ found that a fold change in *BCR‐ABL1* transcript after 1 month could distinguish poor responders, suggesting a possibility of earlier assessment.

In addition, White et al^23^ reported a 36% probability of achieving a 2‐log reduction in low in vitro baseline inhibitory concentration 50% for imatinib (IC50 ≤0.6 μmol/L) by 3 months, suggesting the importance of intrinsic sensitivity to TKIs. Theoretically, *BCR‐ABL1* transcript levels at baseline and at specific time points within 3 months can precisely reflect the in vivo intrinsic sensitivity irrespective of external contributing factors.

Therefore, the present study aimed to identify the prognostic significance of an earlier molecular cutoff and kinetics of *BCR‐ABL1* transcript levels.

## 2 MATERIALS AND METHODS

### 2.1 Patients

The study objectives were (a) to identify the optimal cutoff of *BCR‐ABL1* transcript levels at 4 weeks (very early molecular response; VEMR), (b) to identify predictive factors for the achievement of VEMR, (c) to evaluate the prognostic significance of VEMR, and (d) to evaluate the clinical implication of initial change in *BCR‐ABL1* transcript level within 3 months. In total, 258 patients with CML‐CP, treated with various frontline TKIs (130 treated with imatinib; 128, 2G TKIs) and for whom molecular data were available at 4 weeks (28 ± 3 days), were included in this study. This study included patients from a clinical trial (n = 150) and routine clinical practice (n = 108). To evaluate the optimal VEMR cutoff of *BCR‐ABL1* transcript level and clinical implication of VEMR, data from all 258 patients were prospectively collected. Among them, 183 patients diagnosed at our center had available baseline *BCR‐ABL1* data through retrospective review of clinical records. To evaluate early molecular dynamics and the associated clinical implication, data from 183 patients were analyzed for 4‐week fold change, followed by 4‐week and 3‐month halving time analyses in 156 and 146 patients with no increasing *BCR‐ABL1* transcript level, respectively. The number of patients tested at each time point is presented in Table S1. Patients having minor and atypical *BCR‐ABL1* transcript levels were excluded. Informed consent was obtained from the patients in accordance with the tenets of the Declaration of Helsinki, and all human samples were obtained from the Korea Leukemia Bank, with approval from the institutional review board of the participating institutes.

### 2.2 Cytogenetic and molecular monitoring

Routine cytogenetic analyses were performed using the standard G‐banding method in bone marrow (BM) aspirates, and all cytogenetic responses were estimated on the basis of analyses of more than 20 metaphase cells in a single institution. Cytogenetic responses were monitored at 3‐month intervals until a CCyR was achieved. Molecular responses were monitored using quantitative real‐time polymerase chain reaction (qRT‐PCR) analysis at 3‐month intervals and then at 6‐month intervals after MMRs were achieved. All qRT‐PCR analyses were performed with at least 4.5‐log sensitivity in the central laboratory, and only those qRT‐PCR results with *ABL1* copy numbers greater than 50 000 were analyzed. MMR was defined as a *BCR‐ABL1* transcript level ≤0.1%^IS^. Deep molecular response (DMR) was defined as a reduction in *BCR‐ABL1* transcript levels to 0.0032% or lower on the international scale.

### 2.3 Statistical analysis

OS was measured from the first day of initiation of TKI treatment to any death regardless of cause, and PFS was measured considering progression to the accelerated phase (AP) or blast crisis (BC) as well as death from any cause. OS and PFS were also determined for patients treated with other TKIs after the first‐line TKI administration was discontinued. EFS was divided into (a) EFS‐a, calculated from TKI initiation until any death, progression, treatment failure, and treatment warning (in accordance with the 2013 ELN criteria), and (b) EFS‐b, including EFS‐a, treatment failure with/without frontline TKI discontinuation (except treatment‐free remission), whichever was observed first. Survival curves for OS, PFS, and EFS were plotted using the Kaplan–Meier method and were compared using the log‐rank test. Receiver operating characteristic (ROC) analyses were performed to identify optimal cutoff values of *BCR‐ABL1* transcript at 4 weeks for predicting landmark responses and survival. Potential predictive factors for achieving VEMR were assessed using logistic regression analysis and included age, sex, transcript type, Sokal risk scores, leukocyte count, platelet count, blast %, basophil %, spleen size, hydroxyurea use, type of TKI, CML duration, transcript type, and baseline *BCR‐ABL1* level as a continuous variable. Covariates with a *P*‐value of less than 0.1 in the univariate analyses were added to the multivariate analysis model. Time to events was compared using the log‐rank test with SPSS software (SPSS, Inc.). ROC analyses of fold change and halving time were also performed.

## 3 RESULTS

### 3.1 Patients

Of 258 CML‐CP patients, 130 (50.4%) received imatinib and 128 received 2G TKIs (80 dasatinib, 33 nilotinib, 13 radotinib, and 2 bosutinib). The median age was 43 years (range, 18‐81 years), and 50.8% (n = 131) were female. All patients had major *BCR‐ABL1* transcripts (101 e13a2, 156 e14a2, and 1 e13a2 + e14a2), and the median baseline *BCR‐ABL1* transcript level in 183 patients was 74.6% (range, 4.63‐601.5). Other baseline characteristics including Sokal, Hasford, EUTOS, and ELTS scores are shown in Table 1. Median follow‐up duration was 24 months (range, 1‐182 months), and five patients died (three due to disease progression and two due to non‐CML causes). An additional patient progressed to AP and survived.

**Table 1:** Patient characteristics

Parameters	Total (n = 258)
Age, y	
Median (range)	43 (18‐81)
Sex, number (%)	
Male/female	153 (59.3)/105 (40.7)
Leukocyte count (× 109/L) (NA = 16)	
Median (range)	95.9 (2.82‐532.8)
Platelet (× 10^9^/L) (NA = 6)	
Median (range)	465 (82‐3660)
Blasts (%) (NA = 10)	
Median (range)	1 (0‐14)
Basophils (%) (NA = 13)	
Median (range)	5 (0‐18)
Spleen size, cm (NA = 7)	
Median (range)	3.5 (0‐20)
Sokal risk, number (%)	
Low	88 (34.1)
Intermediate	109 (42.2)
High	59 (22.9)
Unknown	2 (0.8)
Hasford, number (%)	
Low	104 (40.3)
Intermediate	107 (41.5)
High	29 (11.2)
Unknown	18 (7.0)
EUTOS, number (%)	
Low	193 (74.8)
High	45 (17.4)
Unknown	20 (7.8)
ELTS, number (%)	
Low	173 (67.0)
Intermediate	66 (25.6)
High	17 (6.6)
Unknown	2 (0.8)
Transcript type, number (%)	
e13a2	101 (39.1)
e14a2	156 (60.5)
e13a2 + e14a2	1 (0.4)
Baseline *BCR‐ABL1* ^IS^ (%) in 183 patients	
Median (range)	74.6 (4.63‐601.5)
Frontline therapy, number (%)	
Imatinib	130 (50.4)
Nilotinib	33 (12.8)
Dasatinib	80 (31.0)
Bosutinib	2 (0.8)
Radotinib	13 (5.0)
From Dx to TKI treatment in 258 patients, mo	
Median (range)	0.6 (0‐6)
Follow‐up duration, mo	
Median (range)	24 (1‐182)
Outcome	
Alive/death	253/5^a^
Progression	4^b^

a Three disease progression and two non‐CML death.

b Three BC (death) and one AP (alive).

### 3.2 Optimal cutoffs for *BCR‐ABL1* transcript levels at 4 weeks

ROC analysis to identify the optimal cutoff with 4‐week transcript levels allowed us to classify patients as low risk and high risk, with maximal sensitivity and specificity for outcomes, except OS and PFS at specific time points (Table 2). At 4 weeks, patients with transcript levels <41.69% and <40.89% had significantly higher CCyR and MMR rates at 1 year, respectively. The optimal cutoffs for 1‐year MMR achievement were 42.57% and 38.41% in patients treated with frontline imatinib and 2G TKIs, respectively (Tables S2 and S3).

**Table 2:** Relative risk for CCyR, MMR, DMR, and survivals according to the BCR‐ABL1 transcript level at 4 wk

Outcome	Cutoff (%)	No. of patients at risk	RR for transcript level (log)	
			RR (95% CI)	*P*‐value
	BCR‐ABL1 transcript level at 1 mo			
CCyR_1yr				
Low risk	≤41.69	131	1	
High risk	>41.69	127	0.58 (0.44‐0.76)	<0.0001
MMR_1yr				
Low risk	≤40.89	124	1	
High risk	>40.89	134	0.26 (0.17‐0.40)	<0.0001
MMR_5yr				
Low risk	≤39.16	120	1	
High risk	>39.16	138	0.36 (0.25‐0.50)	<0.0001
DMR_1yr				
Low risk	≤30.45	84	1	
High risk	>30.45	174	0.12 (0.04‐0.36)	<0.0001
DMR_5yr				
Low risk	≤33.50	95	1	
High risk	>33.50	163	0.29 (0.16‐0.55)	<0.0001
OS_5yr				
Low risk	≤26.58	59	1	
High risk	>26.58	199	0.21 (0.03‐1.23)	0.083
PFS_5yr				
Low risk	≤26.58	59	1	
High risk	>26.58	199	0.31 (0.06‐1.53)	0.151
EFS_5yr‐a^a^				
Low risk	≤42.75	136	1	
High risk	>42.75	122	2.77 (1.20‐6.38)	0.017
EFS_5yr‐b^b^				
Low risk	≤42.75	136	1	
High risk	>42.75	122	2.17 (1.30‐3.62)	0.003

DMR, deep molecular response; EFS, event‐free survival; MMR, molecular response; OS, overall survival; PFS, progression‐free survival; RR, relative risk; yr, year.

a EFS‐a: PFS + ELN treatment failure + ELN warning.

b EFS‐b: PFS + ELN treatment failure + ELN warning + frontline TKI discontinuation (except treatment free remission).

Furthermore, other cutoffs significantly predicted 5‐year MMR (*P *< 0.0001), 1‐year DMR (*P *<* *0.0001), 5‐year DMR (*P *<* *0.0001), 5‐year EFS‐a (*P *=* *0.017), and 5‐year EFS‐b (*P *=* *0.003). Interestingly, the optimal cutoffs for confidence intervals of 1‐year CCyR (41.69%), 1‐year MMR (40.89%), 5‐year MMR (39.16%), and 5‐year EFS‐a/‐b (42.75%) were similar. However, the cutoffs for CIs of 1‐year DMR (30.45%), 5‐year DMR (33.50%), 5‐year OS (26.58%), and 5‐year PFS (26.58%) were lower.

### 3.3 Rate of VEMR and early molecular responses

**Figure 1:**
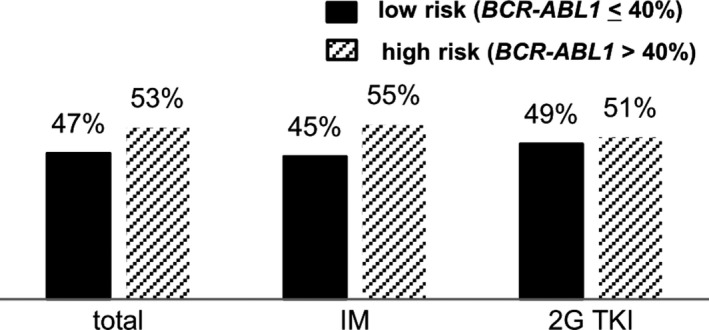
The proportion of low‐risk (*BCR‐ABL1* ≤40%) and high‐risk (*BCR‐ABL1* >40%) patients according to VEMR achievement at 4 wk

Based on the results of ROC analysis, we established a universal cutoff of 40% as VEMR and classified the patients as low risk (BCR‐ABL1 ≤40%) or high risk (BCR‐ABL1 >40%) based on VEMR achievement at 4 weeks. The proportion of the 258 patients achieving VEMR was 47% (121 patients), and VEMR rates between imatinib and 2G TKIs were similar at 45% (58 patients) and 49% (63 patients), respectively (Figure 1).

**Figure 2:**
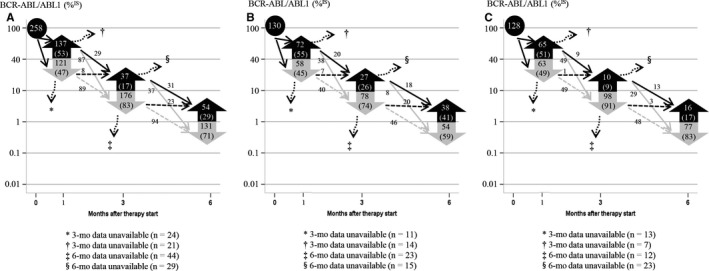
Subsequent change in molecular response. A, total patients; B, imatinib‐treated patients; C, 2G TKI‐treated patients; N (%)

Results of molecular analyses were available for 213 patients at 3 months, and 83% (176 patients) achieved EMR. EMR was achieved in 74% of imatinib‐treated patients and 91% of 2G TKI‐treated patients. At 6 months, molecular analyses were conducted for 185 patients, and EMR was achieved in 71% (131) patients. The EMR rates were 59% and 83% for imatinib‐ and 2G TKI‐treated patients, respectively (Figure 2A‐C).

### 3.4 Subsequent change in molecular response by VEMR

**Figure 3:**
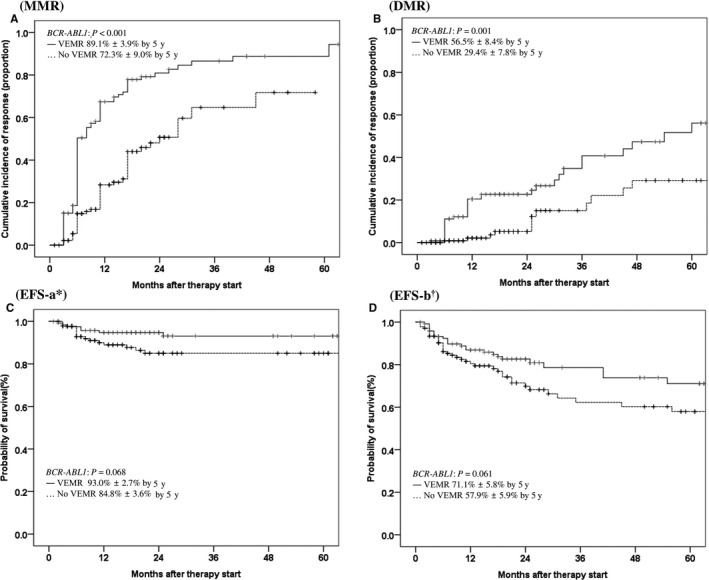
Cumulative incidences of MMR, DMR, and EFS according to VEMR (≤40% and >40%) achievement at 4 wk. VEMR, very early molecular response; MMR, molecular response; DMR, deep molecular response; EFS, event‐free survival; y, year. *EFS‐a: PFS + ELN treatment failure + ELN warning. ^†^ EFS‐b: PFS + ELN treatment failure + ELN warning + frontline TKI discontinuation (except treatment‐free remission)

Among 121 VEMR patients at 4 weeks, results of molecular analyses were available for 97 patients at 3 months and 89 (92%) patients who achieved EMR. Results of molecular analyses were available for 116 of 137 non‐VEMR patients at 3 months, and 75% (87 patients) achieved 3‐month EMR (92% vs 75%; *P *< 0.0001). Of 130 imatinib‐treated patients, results of qRT‐PCR analysis for 3‐month EMR assessment were available for 47 of 58 VEMR patients, and 40 patients (85%) achieved 3‐month EMR. Data were available for 58 of 72 non‐VEMR patients, and 38 patients (66%) achieved 3‐month EMR (85% vs 66%; *P* = 0.026). Of 128 2G TKI‐treated patients, results of molecular analysis for 3‐month EMR assessment were available for 50 of 63 VEMR patients, and 49 patients (98%) achieved 3‐month EMR. Results of molecular analysis were available for 58 of 65 non‐VEMR patients, and 49 patients (84%) achieved 3‐month EMR (Figure 2A‐C) (98% vs 84%; *P* = 0.041). Of 121 VEMR patients, 73 (60.3%) achieved better 12‐month MMR than 21.9% (*P *< 0.001) of 137 non‐VEMR patients (Figure S1). The VEMR cutoff also significantly predicted 5‐year MMR (*P *< 0.001) and 5‐year DMR (*P* = 0.001), and it marginally predicted 5‐year EFS‐a (*P* = 0.068) and 5‐year EFS‐b (*P* = 0.061), but not OS and PFS owing to a small number of events and switching to an alternative TKI (Figure 3).

### 3.5 Outcomes based on 4‐week VEMR and 3‐month EMR

To evaluate the combined clinical significance of *BCR‐ABL1* transcript levels at 4 weeks and 3 months, 213 patients, for whom data for molecular analysis were available at both time points, were classified on the basis of VEMR at 4 weeks (≤ or >40% of *BCR‐ABL1* transcript) and EMR at 3 months (≤ or >10% of *BCR‐ABL1* transcript; Table 3). VEMR + EMR were achieved in 89 patients (group 1); these patients had MMR by 1 year (62.9%) and 5 years (91.7%), and a 5‐year DMR of 62.1%. Five‐year EFS‐a and EFS‐b were achieved in 92.3% and 73.6%, respectively. Twenty‐nine patients had no optimal MRs on both occasions (group 4); these patients had significantly poorer outcomes in terms of 1‐year MMR (6.9%), 5‐year MMR (74.1%), 5‐year DMR (20.0%), 5‐year EFS‐a (70.0%), and EFS‐b (48.2%) than those in group 1. However, there were no differences in 5‐year OS and PFS. Eight patients had VEMR but no EMR (group 2), and 87 patients had EMR but no VEMR (group 3). These patients with discordant *BCR‐ABL1* transcript levels between 4 weeks and 3 months (groups 2 and 3) displayed significant differences in 1‐year CCyR (93.3% vs 77.9%, *P *=* *0.006), 1‐year MMR (62.9% vs 23.2%, *P *<* *0.0001), 5‐year MMR (91.7% vs 64.8%, *P *< 0.0001), 5‐year EFS‐a (92.3% vs 84.0%, *P *=* *0.013), and 5‐year EFS‐b (73.6% vs 56.7%, *P *= 0.016), but no differences in OS (98.8% vs 97.5%, *P *=* *0.603) and PFS (98.8% vs 96.9%, *P *=* *0.389) compared with those in group 1.

**Table 3:** Distribution of patients and outcomes combining *BCR‐ABL1* transcript levels at 4‐wk and 3‐mo

Group	VEMR	3 mos EMR	Number of patients	CCyR by 1 y	MMR by 1 y	CI of MMR by 5 y	CI of DMR by 5 y	OS by 5 y	PFS by 5 y	EFS‐a^a^ by 5 y	EFS‐b^b^ by 5 y
Group 1	Yes	Yes	89	83/89 (93.3%)	56/89 (62.9%)	91.7% ± 4.1	62.1% ± 10.1	98.8% ± 1.2	98.8% ± 1.2	92.3% ± 4.5	73.6% ± 7.1
Group 2		No	8	74/95 (77.9%)	22/95 (23.2%)	64.8% ± 9.7	33.8% ± 10.0	97.5% ± 1.7	96.9% ± 1.8	84.0% ± 4.1	56.7% ± 7.3
Group 3	No	Yes	87								
Group 4		No	29	14/29 (48.3%)	2/29 (6.9%)	74.1% ± 21.5	20.0% ± 17.9	100%	100%	70.0% ± 11.8	48.2% ± 12.8
*Log‐rank P*				<0.0001	<0.0001	<0.0001	0.006	0.723	0.512	0.005	0.007
*Log‐rank P* between subgroups			Group 1 vs 2 and 3	0.006	0.001	<0.0001	0.063	0.603	0.389	0.013	0.016
			Group 1 vs 4	<0.0001	<0.0001	<0.0001	0.015	0.992	0.991	0.005	0.005
			Group 2 and 3 vs 4	0.002	0.121	0.169	0.342	0.971	0.967	0.385	0.285

2G TKI, second‐generation tyrosine kinase inhibition; CI, confidence interval; DMR, deep molecular response; EFS, event‐free survival; MMR, molecular response; OS, overall survival; PFS, progression‐free survival.

a EFS‐a: PFS + ELN treatment failure + ELN warning.

b EFS‐b: PFS + ELN treatment failure + ELN warning + frontline TKI discontinuation (except treatment free remission).

### 3.6 Predictive factors for VEMR and long‐term clinical significance of early molecular dynamics

In univariate analyses of factors affecting VEMR achievement, a *P*‐value of less than 0.1 presented in sex, leukocyte count, platelet count, percentage of blast, percentage of basophil, spleen size, hydroxyurea use, frontline TKI therapy, CML duration, and baseline *BCR‐ABL1* transcript. After adjusting for potential predictive factors, multivariate analyses revealed that low leukocyte count (*P *= 0.008) and frontline 2G TKI therapy (*P *< 0.001) but not baseline *BCR‐ABL1* transcript level (*P *= 0.305) were significantly associated with VEMR achievement (Table S4). However, hydroxyurea use and CML duration were not significantly associated with VEMR achievement and long‐term outcome (Table S5).

As the *BCR‐ABL1* transcript level change within 3 months is a key component in prediction of long‐term outcome, we performed further analyses using baseline transcript level (n = 183), 4‐week transcript level (n = 258), VEMR (n = 258), fold change (n = 183), 4‐week halving time (n = 156), 3‐month halving time (n = 146), and 3‐month EMR (n = 213) in our cohorts. Interestingly, the 4‐week transcript level (≤41.57%), VEMR (≤40%), and 3‐month EMR (≤10%) significantly predicted 12‐month MMR, 5‐year MMR, and 5‐year DMR. The halving time assessed with data from baseline to 3 months more significantly predicted MMR and survival (OS, *P *= 0.031; PFS, *P *= 0.035). However, baseline transcript level, 4‐week fold change, and 4‐week halving time were not predictive for long‐term outcome owing to the direct influence of the baseline *BCR‐ABL1* value calculated with the *ABL1* control gene (Table 4). When the consistency of the clinical significance of the 4‐week *BCR‐ABL1* transcript level in imatinib and 2G TKI cohorts was investigated, as expected, 4‐week *BCR‐ABL1* value significantly predicted MMR in both cohorts, with higher significance in the 2G TKI cohort (Table S6).

**Table 4:** Outcomes according to various *BCR‐ABL1* values between baseline and 3 mo

Parameters	Value	MMR by 12 mo	*P*‐value	MMR by 5 y	*P*‐value	DMR by 5 y	*P*‐value	OS by 5 y	*P*‐value	PFS by 5 y	*P*‐value
Baseline *BCR‐ABL1*	Continuous variable (n = 183)	1.00 (0.99‐1.00)	0.326	1.00 (1.00‐1.00)	0.555	1.00 (0.99‐1.00)	0.394	1.00 (0.99‐1.01)	0.922	1.00 (0.99‐1.01)	0.923
4‐wk *BCR‐ABL1*	Continuous variable (n = 258)	0.98 (0.97‐0.99)	<0.001	0.98 (0.97‐0.99)	<0.001	0.97 (0.95‐0.98)	<0.001	0.99 (0.95‐1.03)	0.596	0.99 (0.97‐1.03)	0.901
4‐wk *BCR‐ABL1*, median	≤41.57% (n = 129)	65.7% ± 4.5	<0.001	88.6% ± 3.9	<0.001	54.7% ± 8.0	0.002	97.5% ± 1.4	0.679	97.6% ± 1.4	0.996
	>41.57% (n = 129)	29.1% ± 4.8		71.4% ± 9.3		28.7% ± 8.1		97.1% ± 2.2		96.3% ± 2.3	
VEMR	≤40% (n = 121)	67.9% ± 4.6	<0.001	89.1% ± 3.9	<0.001	56.5% ± 8.4	0.001	97.3% ± 1.5	0.581	97.4% ± 1.5	0.873
	>40% (n = 137)	29.0% ± 4.6		72.3% ± 9.0		29.4% ± 7.8		97.2% ± 2.1		96.5% ± 2.2	
3‐mo EMR	≤10% (n = 176)	52.5% ± 4.2	<0.001	85.2% ± 5.1	<0.001	50.6% ± 7.5	0.041	98.8% ± 0.9	0.478	98.2% ± 1.0	0.692
	>10% (n = 37)	8.5% ± 5.9		47.6% ± 18.8		16.7% ± 15.2		96.8% ± 3.2		97.1% ± 2.8	
4‐wk FC	FC≤1 (n = 156)	47.3% ± 4.3	0.899	82.7% ± 5.1	0.485	43.9% ± 7.3	0.433	97.9% ± 1.2	0.464	98.0% ± 1.1	0.466
	FC>1 (n = 27)	44.5% ± 10.1		63.0% ± 13.4		33.4% ± 15.7		100%		100%	
4‐wk HT^a^	≤22 d (n = 53)	39.8% ± 7.4	0.209	79.5% ± 8.8	0.292	38.6% ± 13.4	0.450	95.8% ± 2.9	0.231	96.1% ± 2.7	0.214
	>22 d (n = 103)	50.8% ± 5.3		83.7% ± 6.1		46.1% ± 8.7		99.0% ± 1.0		99.0% ± 1.0	
3‐mo HT^b^	≤21 d (n = 101)	52.4% ± 5.4	0.015	82.1% ± 6.9	0.039	46.2% ± 8.2	0.097	100%	0.031	100%	0.035
	>21 d (n = 45)	35.4% ± 7.7		62.1% ± 9.5		37.7% ± 18.2		94.9% ± 3.5		95.4% ± 3.2	

DMR, deep molecular response; FC, fold change; HT, halving time; MMR, molecular response; OS, overall survival; PFS, progression‐free survival; VEMR, very early molecular response.

a Twenty‐seven patients with increasing transcript level were excluded in this analysis.

b Additional 10 patients with increasing transcript level during this period were excluded in this analysis.

## 4 DISCUSSION

In the TKI era, early molecular response was first reported to predict short‐term outcomes such as MCyR with *BCR‐ABL1* transcript levels reducing to 20% and 50% of baseline within 2 months and 4 weeks of imatinib initiation, respectively.^24^, ^25^

Thereafter, several studies involving frontline TKIs for CML‐CP patients reported that EMRs at 3 and 6 months strongly predict long‐term responses and survival.^4^, ^15^, ^26^

Three‐month EMR was previously predicted to be achieved in 50%‐71% of imatinib‐treated new CP patients and in 75%‐91% of frontline 2G TKI‐treated patients. In addition, 6‐month EMR (*BCR‐ABL1* transcript level ≤1%) was predicted to be achieved in 49%‐58% of imatinib‐treated patients and in 69%‐82% of 2G TKI‐treated patients.^14^, ^27^, ^28^

In the present study, of 213 patients eligible for 3‐month molecular analysis, 83% (176 patients) achieved EMR at 3 months and 71% (results of molecular analysis available for 131 of 185 patients) achieved EMR at 6 months. Among 130 frontline imatinib‐treated patients, the EMR rates at 3 and 6 months were 74% and 59%, respectively. Of 128 patients administered frontline 2G TKIs, 91% and 83% achieved EMR at 3 and 6 months, respectively. In this study, EMR rates were comparable with those of DASISION, ENESTnd, RERISE, and BFORE studies,^13^, ^14^, ^27^, ^28^ indicating the use of homogeneous approaches in terms of molecular analysis and study population. Interestingly, the optimal cutoffs for higher CCyR, MMR, and EFS were similar (approximately 40%), which was albeit as a VEMR. However, DMR, OS, and PFS were different (26.58%‐33.50%) owing to rare events. In addition, the optimal cutoff for DMR prediction could be determined in only the 2G TKI‐treated patients (Tables S2 and S3). Hence, early assessment of *BCR‐ABL1* transcript levels has a better predictive power for deeper response with more potent TKI.

With VEMR defined as 40% of *BCR‐ABL1* transcript levels, the proportion of patients achieving VEMR was 47% of all 258 patients. VEMR predicted subsequent 3‐month EMR, and the 3‐month EMR rate was higher in 2G TKI‐treated patients (98% vs 85%). Of patients who failed to achieve VEMR, 84% and 66% achieved 3‐month EMR among those administered 2G TKI and imatinib, respectively. Our results suggest that more potent TKI can maintain optimal molecular response and may better rescue patients with VEMR failure. Regardless of the variety of first‐line TKI used, VEMR significantly predicted the cumulative MMR and DMR, and EFS was marginally predicted.

In our previous study, patients who achieved EMR both at 3 and 6 months had better outcomes than those achieving no EMR on both occasions. Moreover, patients with discordant *BCR‐ABL1* transcript levels between 3 and 6 months also showed poor outcomes.^17^ Therefore, we analyzed the clinical significance of combining 4‐week VEMR and 3‐month EMR. In our study, 89 patients who achieved VEMR and 3‐month EMR showed a significant predictive power for landmark responses and survival such as CCyR, MMR, DMR, and EFS. Patients who showed discordant *BCR‐ABL1* transcript levels between 4 weeks and 3 months (groups 2 and 3) displayed similar poor responses and outcomes compared with those who failed to display optimal responses at two occasions of 4 weeks and 3 months (group 4). This finding suggests that the additional molecular analysis at early time points may improve the predictive accuracy and may be a powerful predictor of subsequent long‐term outcomes.

Hanfstein et al^10^ and Branford et al^29^ reported that an initial decline in *BCR‐ABL1* transcript levels was a critical predictor for poor outcomes. In addition, leukocyte count, blast count, risk score, spleen size, lactate dehydrogenase levels, achievement of CHR, TKI dose intensity, and blood level of TKI were significantly associated with 3‐month EMR achievement.^13^, ^17^, ^30^ Therefore, the factors influencing VEMR achievement need to be further evaluated. In the present study, low leukocyte count and frontline 2G TKI therapy were significantly associated with VEMR achievement.

Recently, El Missiry et al^22^ suggested that an initial decline in *BCR‐ABL1* transcript level determined using the *GUS* control gene after 1 month (as a fold change) may distinguish early responders based on disease biology. However, analysis of our cohort without interruption or dose reduction of TKI treatment during the first 4 weeks did not reveal any associations between 4‐week fold change, halving time, and further molecular responses, suggesting that assessment of earlier molecular dynamics with baseline *BCR‐ABL1* transcript level as assessed by the *ABL1* control gene is considerably limited. In contrast, the 4‐week transcript level (as a continuous variable and with median value of 41.57%), VEMR (≤40%), and 3‐month EMR (≤10%) significantly predicted further molecular responses including 5‐year MMR and DMR, with higher significance in the 2G TKI cohort. Moreover, the halving time to 3 months significantly predicted outcome. Consistent with our findings, Branford et al^18^ found that the 3‐month halving time may provide a significant predictive power to distinguish responders. As the initial dosage of TKIs was maintained for the majority of our patients by the first qRT‐PCR assay, the 4‐week *BCR‐ABL1* transcript levels of individual patients may reflect the true cellular sensitivity to TKI. In addition, identifying very‐low‐risk patients, considering baseline biological factors, may be critical for affordable treatment and further treatment‐free remission trials.

This study involved subgroup analyses of a heterogeneous population, suggesting a limitation in interpretation.

However, this study supports the possibility of earlier assessment using the 4‐week transcript levels including VEMR. To confirm the further clinical benefit of VEMR and an early switch of therapy, a randomized prospective trial in a larger homogeneous population with longer follow‐up is warranted.

In conclusion, we have determined the clinical significance of the 4‐week transcript levels including VEMR and the predictive factors associated with its achievement, which were associated with intrinsic in vivo sensitivity to TKI. Based on our results, as a powerful predictor of molecular landmark responses including DMR, a change in *BCR‐ABL1* transcript within 4 weeks and VEMR assessment might be useful to identify patients potentially eligible for treatment‐free remission.

## CONFLICT OF INTEREST

None declared.
