# Supplementary figures and images for: BCR‐ABL1 transcript levels at 4 weeks have prognostic significance for time‐specific responses and for predicting survival in chronic‐phase chronic myeloid leukemia patients treated with various tyrosine kinase inhibitors
Source: Cancer Med. 2018 Aug 31;7(10):5107–17. doi: 10.1002/cam4.1753 (PMC6198233; doi:10.1002/cam4.1753)

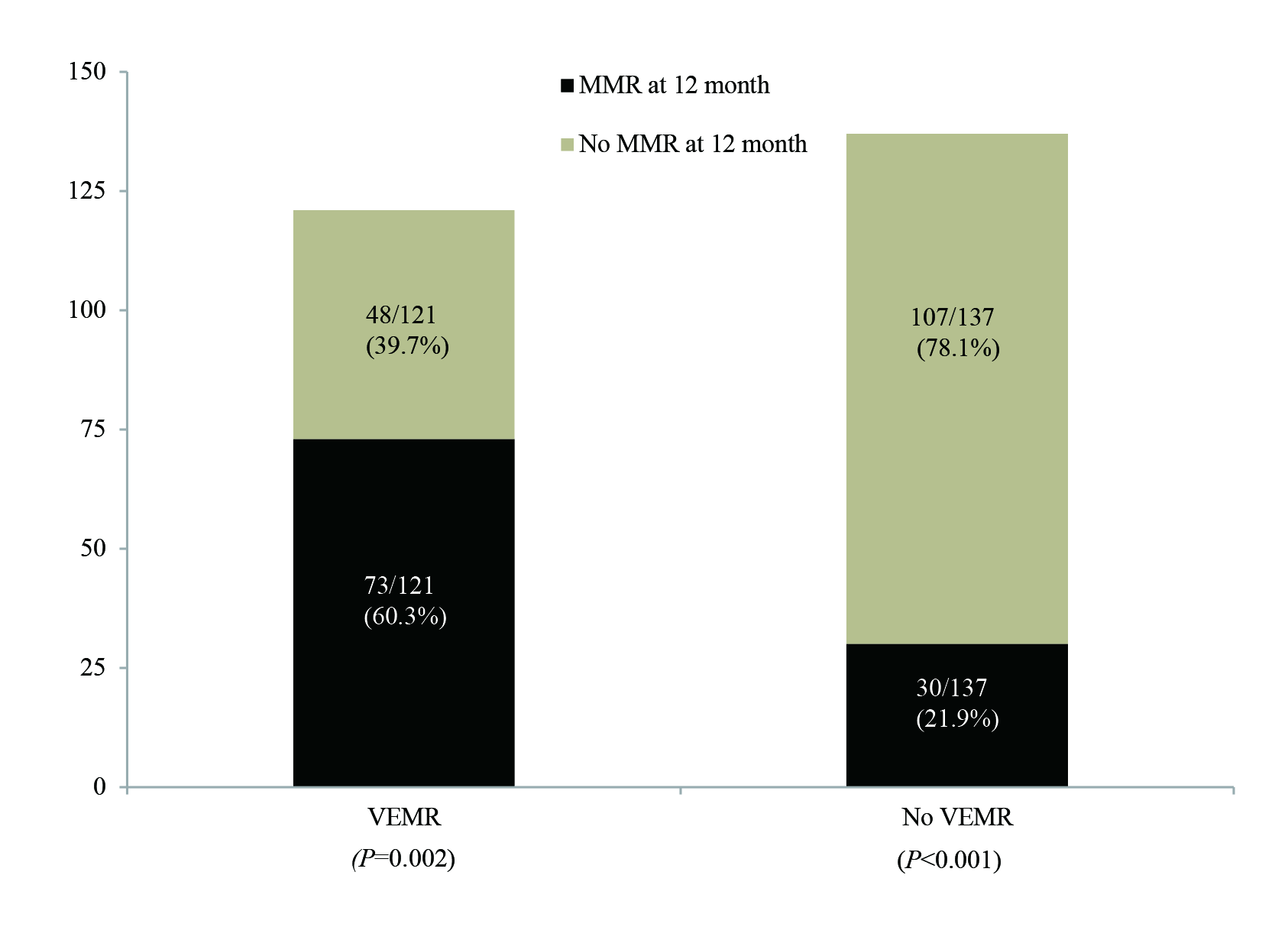

Supplement: Supplementary file 1 [file CAM4-7-5107-s001.tif]
