# Supplementary material for: BCR‐ABL1 transcript levels at 4 weeks have prognostic significance for time‐specific responses and for predicting survival in chronic‐phase chronic myeloid leukemia patients treated with various tyrosine kinase inhibitors
Source: Cancer Med. 2018 Aug 31;7(10):5107–17. doi: 10.1002/cam4.1753 (PMC6198233; doi:10.1002/cam4.1753)
Supplement: Supplementary file 2 [file CAM4-7-5107-s002.docx]

**Supporting information Tables and Figures**

**Supporting Information Table 1. Patients who were available molecular data at each time points**

| **At baseline** | **1 mos (n=258)** | **3 mos (n=213)** | **6 mos (n=185)** | **12 mos (n=183)** | **IM (n=130)** | **2G (n=128)** | **Total (n=258)** |
| --- | --- | --- | --- | --- | --- | --- | --- |
| Yes (n=183) | o | o | o | o | 42 | 50 | 92 |
|  | o | o | o | - | 14 | 9 | 23 |
|  | o | o | - | o | 13 | 8 | 21 |
|  | o | - | o | o | 9 | 15 | 24 |
|  | o | o | - | - | 4 | 8 | 12 |
|  | o | - | o | - | 3 | 0 | 3 |
|  | o | - | - | o | 4 | 1 | 5 |
|  | o | - | - | - | 1 | 2 | 3 |
| No (n=75) | o | o | o | o | 15 | 9 | 24 |
|  | o | o | o | - | 3 | 10 | 13 |
|  | o | - | o | o | 5 | 0 | 5 |
|  | o | o | - | o | 9 | 2 | 11 |
|  | o | o | - | - | 5 | 12 | 17 |
|  | o | - | o | - | 1 | 0 | 1 |
|  | o | - | - | o | 0 | 1 | 1 |
|  | o | - | - | - | 2 | 1 | 3 |

**Supporting Information Table 2. Relative risks of outcomes according to ROC cutoffs of 4-week *BCR-ABL1* transcript level in imatinib group (n=130)**

| **Outcome** | **Cutoff (%)**  ***BCR-ABL1* transcript level at 4 weeks** | **No. of patients at risk** | **Relative risk (95% CI)** | ***P*-value** |
| --- | --- | --- | --- | --- |
| CCyR by 1yr |  |  |  |  |
| Low risk | ≤38.53 | 57 | 1 |  |
| High risk | >38.53 | 73 | 0.63(0.43-0.94) | 0.023 |
| MMR by 1yr |  |  |  |  |
| Low risk | ≤42.57 | 63 | 1 |  |
| High risk | >42.57 | 67 | 0.40(0.21-0.74) | 0.004 |
| MMR by 5yr |  |  |  |  |
| Low risk | ≤42.57 | 63 | 1 |  |
| High risk | >42.57 | 67 | 0.50(0.31-0.81) | 0.005 |
| DMR by 1yr |  |  |  |  |
| Low risk | ≤26.57 | 28 | 1 |  |
| High risk | >26.57 | 102 | 0.07(0.01-0.55) | 0.012 |
| DMR by 5yr |  |  |  |  |
| Low risk | ≤43.70 | 69 | 1 |  |
| High risk | >43.70 | 61 | 0.31(0.12-0.83) | 0.019 |
| OS by 5yr* |  |  |  |  |
| Low risk | ≤26.59 | 29 | 1 |  |
| High risk | >26.59 | 101 | 0.29(0.04-2.07) | 0.218 |
| PFS by 5yr* |  |  |  |  |
| Low risk | ≤70.25 | 110 | 1 |  |
| High risk | >70.25 | 20 | 3.61(0.60-21.62) | 0.161 |
| EFS-a by 5yr^†^ |  |  |  |  |
| Low risk | ≤49.45 | 81 | 1 |  |
| High risk | >49.45 | 49 | 2.75(1.19-6.35) | 0.018 |
| EFS-b by 5yr^‡^ |  |  |  |  |
| Low risk | ≤42.75 | 64 | 1 |  |
| High risk | >42.75 | 66 | 1.87(1.07-3.27) | 0.027 |

Abbreviations: RR, relative risk; CI, confidence interval; 2G TKI, 2^nd^ generation tyrosine kinase inhibition; OS, overall survival; PFS, progression-free survival; EFS, event-free survival

* OS, PFS in 2G TKI group; death (n=0), progression (n=0); no calculation.

^†^ EFS-a : PFS + ELN treatment failure + ELN warning

^‡^ EFS-b : PFS + ELN treatment failure + ELN warning + frontline TKI discontinuation (except treatment free remission)

**Supporting Information Table 3. Relative risks of outcomes according to ROC cutoffs of 4-week *BCR-ABL1* transcript level in 2G TKI group (n=128)**

| **Outcome** | **Cutoff (%)**  ***BCR-ABL1* transcript level at 4 weeks** | **No. of patients at risk** | **Relative risk (95% CI)** | ***P*-value** |
| --- | --- | --- | --- | --- |
| CCyR by 1yr |  |  |  |  |
| Low risk | ≤47.86 | 80 | 1 |  |
| High risk | >47.86 | 48 | 0.68(0.45-1.01) | 0.056 |
| MMR by 1yr |  |  |  |  |
| Low risk | ≤38.41 | 60 | 1 |  |
| High risk | >38.41 | 68 | 0.14(0.07-0.27) | <.0001 |
| MMR by 5yr |  |  |  |  |
| Low risk | ≤38.51 | 61 | 1 |  |
| High risk | >38.51 | 67 | 0.23(0.13-0.39) | <.0001 |
| DMR by 1yr |  |  |  |  |
| Low risk | ≤30.45 | 44 | 1 |  |
| High risk | >30.45 | 84 | 0.13(0.04-0.47) | 0.002 |
| DMR by 5yr |  |  |  |  |
| Low risk | ≤30.45 | 44 | 1 |  |
| High risk | >30.45 | 84 | 0.16(0.05-0.46) | 0.001 |
| EFS-a by 5yr* |  |  |  |  |
| Low risk | ≤14.96 | 7 | 1 |  |
| High risk | >14.96 | 121 | 0.10(0.01-1.09) | 0.059 |
| EFS-b by 5yr^†^ |  |  |  |  |
| Low risk | ≤14.96 | 7 | 1 |  |
| High risk | >14.96 | 121 | 0.18(0.04-0.91) | 0.037 |

Abbreviations: RR, relative risk; CI, confidence interval; 2G TKI, 2^nd^ generation tyrosine kinase inhibition; OS, overall survival; PFS, progression-free survival; EFS, event-free survival

* EFS-a : PFS + ELN treatment failure + ELN warning

^†^ EFS-b : PFS + ELN treatment failure + ELN warning + frontline TKI discontinuation (except treatment free remission)

**Supporting Information Table 4. Univariate and multivariate analyses of variables affecting VEMR achievement**

| **Variables** | **Number** | **Univariate analyses** | | **Multivariate analyses** | |
| --- | --- | --- | --- | --- | --- |
|  |  | **RR(95% CI)** | ***P*-value** | **RR(95% CI)** | ***P*-value** |
| Age (yrs): median - 43 (18-81)* | 258 | 1.01 (0.99-1.02) | 0.268 | - | - |
|  |  |  |  |  |  |
| Sex |  |  |  |  |  |
| male | 153 | 1 |  |  |  |
| female | 105 | 1.43 (1.00-2.05) | 0.052 | 1.10 (0.67-4.70) | 0.709 |
|  |  |  |  |  |  |
| Sokal risk |  |  |  |  |  |
| low/intermediate | 197 | 1 |  | - | - |
| high | 59 | 0.92 (0.58-1.45) | 0.728 | - | - |
| NA | 2 | - | - | - | - |
|  |  |  |  |  |  |
| Complete blood counts |  |  |  |  |  |
| Leukocyte count, x 10^9^* | 242 | 0.99 (0.99-1.00) | <0.001 | 0.99 (0.99-1.00) | 0.008 |
| Platelet count, x 10^9^* | 252 | 1.00 (1.00-1.00) | 0.007 | 1.00 (1.00-1.00) | 0.857 |
| Percentage of blast, %* | 248 | 0.90 (0.80-1.01) | 0.072 | 1.11 (0.97-1.29) | 0.136 |
| Percentage of eosinophil, %* | 248 | 1.04 (0.97-1.12) | 0.907 | - | - |
| Percentage of basophil, %* | 245 | 1.04 (1.00-1.08) | 0.083 | 1.06 (0.99-1.12) | 0.092 |
|  |  |  |  |  |  |
| Spleen size from costal margin, cm* | 241 | 0.88 (0.83-0.93) | <0.001 | 0.93 (0.85-1.02) | 0.120 |
|  |  |  |  |  |  |
| Hydroxyurea |  |  |  |  |  |
| yes | 160 | 1 |  |  |  |
| no | 98 | 1.07 (0.75-1.54) | 0.714 | - | - |
|  |  |  |  |  |  |
| Hydroxyurea duration, day* | 258 | 0.99 (0.97-1.01) | 0.268 | - | - |
|  |  |  |  |  |  |
| Frontline TKI therapy |  |  |  |  |  |
| imatinib | 130 | 1 |  |  |  |
| 2G TKI | 128 | 1.79 (1.25-2.58) | 0.002 | 2.77 (1.64-4.70) | <0.001 |
|  |  |  |  |  |  |
| Time from Dx to TKI initiation (mos)* | 258 | 1.11 (0.92-1.32) | 0.273 | - | - |
|  |  |  |  |  |  |
| Time from Dx to TKI initiation (mos): median - 0.6 |  |  |  |  |  |
| 0-1 | 222 | 1 |  |  |  |
| 1-3 | 30 | 1.46 (0.89-2.40) | 0.129 | - | - |
| 3-6 | 6 | 2.31 (1.01-5.28) | 0.046 | 1.62 (0.43-6.14) | 0.480 |
|  |  |  |  |  |  |
| Transcript type |  |  |  |  |  |
| e13a2 | 101 | 1 |  |  |  |
| e14a2 | 156 | 1.08 (0.75-1.60) | 0.656 | - | - |
| e13a2+e14a2 | 1 | - | 0.965 | - | - |
|  |  |  |  |  |  |
| Baseline *BCR-ABL1* transcript, %* | 183 | 0.99 (0.99-1.00) | 0.030 | 1.00 (1.00-1.01) | 0.305 |

* continuous variable

**Supporting Information Table** **5. Clinical impact of CML duration and hydroxyurea use before TKI initiation**

| **Parameters** | **Value** | **VEMR** | ***P*-value** | **3mos EMR** | ***P*-value** | **MMR**  **by 12 mos** | ***P*-value** | **MMR**  **by 5yrs** | ***P*-value** | **OS**  **by 5yrs** | ***P*-value** | **PFS**  **by 5yrs** | ***P*-value** |
| --- | --- | --- | --- | --- | --- | --- | --- | --- | --- | --- | --- | --- | --- |
| CML  duration | continuous variable (n=258) | 1.11 (0.92-1.32) | 0.273 | 0.97 (0.74-1.26) | 0.796 | 1.11 (0.87-1.41) | 0.401 | 1.08 (0.89-1.32) | 0.435 | 1.14 (0.44-2.91) | 0.786 | 1.00 (0.35-2.87) | 0.999 |
| CML  duration | 0-1 mos (n=222)  1-3 mos (n=30)  3-6 mos (n=6) | 1  1.46 (0.89-2.40)  2.31 (1.01-5.28) | 0.129  0.046 | 1  0.90 (0.55-1.47)  1.20 (0.38-3.76) | 0.672  0.754 | 1  1.45 (0.82-2.55)  1.39 (0.44-4.41) | 0.198  0.572 | 1  1.33 (0.78-2.29)  1.28 (0.51-3.23) | 0.295  0.600 | 1  5.16 (0.86-30.92)  - | 0.072  0.991 | 1  3.83 (0.70-20.91)  - | 0.121  0.989 |
| HUR  duration | continuous variable  (n=258) | 0.99 (0.97-1.01) | 0.268 | 1.00 (0.98-1.01) | 0.599 | 1.00 (0.98-1.02) | 0.861 | 1.00 (0.98-1.02) | 0.994 | 0.92 (0.80-1.07) | 0.281 | 0.90 (0.78-1.04) | 0.166 |
| HUR | yes (n=160)  no (n=98) | 1  1.07 (0.75-1.53) | 0.714 | 1  1.07 (0.79-1.45) | 0.652 | 1  0.77 (0.52-1.16) | 0.218 | 1  0.87 (0.62-1.24) | 0.456 | 1  1.82 (0.29-11.5) | 0.523 | 1  2.69 (0.48-15.23) | 0.263 |

**Supporting Information Table 6. Comparison of TKIs on outcomes according to *BCR-ABL1* transcript level at 4 weeks**

**A. Imatinib group (n=130)**

| **Parameters** | **Value** | **MMR**  **by 12 mos** | ***P*-value** | **MMR**  **by 5yrs** | ***P*-value** | **OS**  **by 5yrs** | ***P*-value** | **PFS**  **by 5yrs** | ***P*-value** |
| --- | --- | --- | --- | --- | --- | --- | --- | --- | --- |
| 4-week *BCR-ABL1,* median | <43.07% (n=65)  >43.07% (n=65) | 47.6%±6.5  27.6%±6.1 | 0.005 | 81.9%±6.3  71.5%±9.6 | 0.007 | 96.9%±2.2  96.3%±2.6 | 0.989 | 96.9%±1.5  94.8%±3.0 | 0.651 |
| VEMR | <40% (n=58)  >40% (n=72) | 45.9%±6.8  31.1%±6.0 | 0.028 | 81.2%±6.6  73.3%±9.0 | 0.037 | 96.5%±2.4  96.6%±2.4 | 0.836 | 96.6%±2.4  95.3%±2.7 | 0.834 |

**B. 2G TKI group (n=128)**

| **Parameters** | **Value** | **MMR**  **by 12 mos** | ***P*-value** | **MMR**  **by 5yrs** | ***P*-value** | **OS**  **by 5yrs** | ***P*-value** | **PFS**  **by 5yrs** | ***P*-value** |
| --- | --- | --- | --- | --- | --- | --- | --- | --- | --- |
| 4-week *BCR-ABL1,* median | <40.80% (n=64)  >40.80% (n=64) | 89.1%±4.5  23.8%±6.6 | <0.001 | 93.5%±3.6  50.1%±12.3 | <0.001 | 98.2%±1.8  100% | 0.345 | 98.4%±1.6  100% | 0.313 |
| VEMR | <40% (n=63)  >40% (n=65) | 91.0%±4.2  23.3%±6.5 | <0.001 | 95.5%±3.1  47.4%±11.5 | <0.001 | 98.2%±1.8  100% | 0.336 | 98.4%±1.6  100% | 0.306 |
